# Recent Advances in Research on Antibacterial Metals and Alloys as Implant Materials

**DOI:** 10.3389/fcimb.2021.693939

**Published:** 2021-07-02

**Authors:** Juyang Jiao, Shutao Zhang, Xinhua Qu, Bing Yue

**Affiliations:** Department of Bone and Joint Surgery, Department of Orthopaedics, Renji Hospital, School of Medicine, Shanghai Jiao Tong University, Shanghai, China

**Keywords:** implant-associated infection, antibacterial metals and alloys, titanium alloy, cobalt alloy, degradable metal and alloy, tantalum

## Abstract

Implants are widely used in orthopedic surgery and are gaining attention of late. However, their use is restricted by implant-associated infections (IAI), which represent one of the most serious and dangerous complications of implant surgeries. Various strategies have been developed to prevent and treat IAI, among which the closest to clinical translation is designing metal materials with antibacterial functions by alloying methods based on existing materials, including titanium, cobalt, tantalum, and biodegradable metals. This review first discusses the complex interaction between bacteria, host cells, and materials in IAI and the mechanisms underlying the antibacterial effects of biomedical metals and alloys. Then, their applications for the prevention and treatment of IAI are highlighted. Finally, new insights into their clinical translation are provided. This review also provides suggestions for further development of antibacterial metals and alloys.

## Introduction

Orthopedic implants, including fracture fixation devices, artificial prostheses that replace joints and intervertebral discs, and bone defect fillers, help maintain, support, and restore the structure and function of the musculoskeletal system (Wang and Tang, 2019). Implant materials commonly used in orthopedics include metals, alloys, ceramics, and polymers, among which metals and alloys occupy the main position. When left in the body for a long time, these implants inevitably increase the risk of infection (Izakovicova et al., 2019). Particularly, implant-associated infections (IAI) are one of the most common and serious complications in orthopedics and include fracture-related infection and prosthetic joint infection (PJI). Fracture-related infections occur in 1–2% of closed fractures following internal fixation and up to 30% of open fractures (Kuehl et al., 2019), whereas PJI has an incidence of 0.5–2% and 10–15% in primary and revision joint replacements, respectively (Mortazavi et al., 2010; Kapadia et al., 2016). The incidence of spinal device-related infections is 0.5–10% (Margaryan et al., 2020). IAI can lead to implant failure, which usually requires more complicated and lengthy treatment, including the use of large amounts of antibiotics and multiple surgical interventions; it is accompanied by poor prognosis. In addition to high morbidity and mortality, IAI cause a large economic burden on the medical system and society. In the United States, the average cost of treating PJI of the hip or knee is approximately $100,000 per individual; by 2030, the economic burden of PJI related to the hip and knee is estimated to reach $1.85 billion (Premkumar et al., 2020).

IAI usually involves intricate interactions between bacteria, host cells, and implant materials. Under normal circumstances, osseointegration occurs between the implant interface and the surrounding bone tissue cells to promote bone ingrowth and healing. However, the implant itself is a good substrate for bacterial adhesion and biofilm formation, leading to the formation of a local immunosuppressive niche, which is more likely to cause bacterial adhesion and infection. Once the pathogenic bacteria enter around the implant, they compete with the bone cells for the surface, adhere to and colonize the implant surface, and form a biofilm to further resist the effects of antibiotics and the host immune response (Arciola et al., 2018). The most common pathogenic bacteria in orthopedic IAI are gram-positive bacteria, including *Staphylococcus aureus* and coagulase-negative staphylococci (e.g., *Staphylococcus epidermidis* and *Staphylococcus saprophyticus*), accounting for approximately 50–60% of PJI; *Streptococcus* and *Faecococci* account for approximately 10%. Meanwhile, IAI cases caused by aerobic gram-negative bacteria (e.g., *Escherichia coli* and *Pseudomonas aeruginosa*), anaerobic gram-negative bacteria (e.g., *Propionibacterium acnes*, *Bacteroides fragilis*), and fungi are increasing (Tande and Patel, 2014; Kapadia et al., 2016).

Antibiotics are the most effective means of preventing and treating infections in the clinical setting. Nonetheless, bacterial resistance to antibiotics is steadily increasing owing to their irregular or excessive use. Therefore, the emergence of multi-drug resistant bacteria, such as methicillin-resistant *S. aureus* (MRSA) and methicillin-resistant *S. epidermidis* (MRSE) makes the treatment of IAI more challenging (Li and Webster, 2018). Thus, there is an urgent need to find new alternatives to combat drug-resistant bacteria and biofilms.

Designing implants with antibacterial properties, especially reducing bacterial adhesion and inhibiting biofilm formation, is one of the most promising strategies to prevent and treat IAI. Coating, surface modification, and metal alloying are the most commonly used modification methods for implants. The coating forms an additional layer by loading or diffusing substances on the surface of the substrate, whereas surface modification refers to the modification of the thin layer on the surface of the substrate at the atomic, molecular, or geomorphological level (Premkumar et al., 2020). These two methods do not damage the overall performance of the material and only perform specific functions to achieve bacteriostatic or bactericidal characteristics; however, they are associated with limitations such as limited antibacterial time and coating delamination. The loaded antimicrobial arsenal includes antimicrobial metal elements, lysozymes, antimicrobial peptides, monoclonal antibodies, bacteriophages, and probiotics (Cloutier et al., 2015). In recent years, many reviews have discussed this issue with surface modification in detail (Cloutier et al., 2015; Raphel et al., 2016; Ahmadabadi et al., 2020). Meanwhile, unlike coating and surface modification, metal alloying involves the addition of an appropriate amount of antibacterial metal elements to the substrate material, thus transforming it into an alloy material with antibacterial ability (i.e., antibacterial metals and alloys), through specific metal processing (Ren and Yang, 2017). Antibacterial metals and alloys have been extensively studied owing to their long-lasting antibacterial properties, easy control, and convenient preparation methods.

This review summarizes the latest applications of antimicrobial metals and alloys for the prevention and treatment of orthopedic IAI and recent developments for their fabrication. First, we discuss the complex interactions between bacteria, materials, and host cells during IAI and the potential mechanisms of antibacterial metals and alloys. Then, we enumerate the recent applications of different antibacterial metal materials in orthopedics, including antibacterial titanium (Ti) alloys, antibacterial cobalt (Co) alloys, biodegradable antibacterial metals and alloys, and antibacterial tantalum (Ta). Finally, new strategies to expand the clinical applications of antibacterial metals and alloys in orthopedics are proposed.

## Intricate Interactions Between Bacteria, Implant Materials, and Host Cells in IAI

### Bacteria and Implant Materials

#### Bacterial Adhesion

Bacterial adhesion is the first step in IAI, paving the way for subsequent colonization. Compared with the surrounding aqueous environment, bacteria prefer to adhere to the surface of the material. The first stage of bacterial adhesion on the implant surface involves the initial, non-specific, reversible physical and chemical attachment, whereas the second stage involves the specific, irreversible molecular and cell attachment (Filipovic et al., 2020).

Bacteria are initially attracted to the material surface by physical forces, such as Brownian motion, gravitational forces, Lifshitz–van der Waals attraction forces, surface electrostatic charges, and hydrophobic interactions. This is followed by cell adsorption and cell attachment (Katsikogianni and Missirlis, 2004). Physical forces can be divided into two categories according to the distance between the bacteria and the surface: long-range (>50 nm) and short-range interactions (<5 nm, including chemical bonding, ionic and dipole interactions, and hydrophobic interactions) (Khalid et al., 2020). The extended Derjaguin–Landau–Verwey–Overbeek theory (xDLVO theory) is often used to predict the initial adhesion interaction between bacteria and the surface of materials. The force between bacteria and the surface is the vector sum of Lifshitz–van der Waals, electrical double layer, and Lewis acid–base forces (Linklater et al., 2021).

The second stage is the close and irreversible combination of bacterial structures, such as flagella, capsules, and nanofibers, with non-biological surfaces. In addition, the surface of the bare material is quickly encapsulated by extracellular matrix (ECM) proteins in the body. Adhesins, such as microbial surface components recognizing adhesive matrix molecules (MSCRAMMs), can mediate the binding of the bacteria and ECM proteins on the coating surface. Adhesin-mediated interactions cause bacteria to aggregate. Bacterial adhesion is a complex process that is affected by bacterial characteristics, material surface characteristics, environmental factors, and hydrodynamic factors (Filipovic et al., 2020).

#### Biofilm Formation

Biofilm formation is a dynamic process that mainly comprises the following stages: bacterial adhesion, microcolony formation, biofilm maturation, and biofilm dissipation (Zhang et al., 2020b). The individual sparsely distributed bacterial cells attached to the surface gradually gather to form small colonies and secrete extracellular polymer matrix (EPS) to surround bacterial colonies. The biofilm gradually matures *via* the signal system to form a tower-like structure and a subsequent tighter attachment with the material (Arciola et al., 2018). Biofilm formation provides a protective 3D structure for the bacteria. The impermeability of the biofilm network, horizontal gene transfer, and phenotypic differences among the multiple microbes in the matrix are the main factors responsible for their enhanced resistance to conventional antibiotics and host immunity (Makabenta et al., 2021). EPS is mainly composed of extracellular polysaccharides, proteins, extracellular DNA (eDNA), and teichoic acid (Flemming et al., 2016). In *S. aureus* and *S. epidermidis*, the expression of polysaccharide intercellular adhesion (PIA) and the release of eDNA are vital mechanisms of biofilm formation. PIA is the main component of the extracellular polysaccharide matrix, and its expression is related to the *icaA/D/B/C* locus (Arciola et al., 2015). In an unfavorable environment, some bacterial cells sacrifice themselves to provide a suitable living environment for the surviving bacteria. These autolyzed bacteria release eDNA and mediate the maturation and stabilization of the biofilm matrix (Okshevsky and Meyer, 2015). Mature biofilms dissipate through extracellular enzymes and phenol-soluble modulins, which cause bacteria to resume a planktonic state and start a new cycle in neighboring locations (Le et al., 2014).

### Bacteria and Host Cells

In orthopedic IAI, pathogenic bacteria enter the prosthesis site through blood-borne and contiguous spread (Kavanagh et al., 2018). Innate immune cells, including neutrophils and macrophages, are recruited to the infection site by pattern recognition receptors, such as Toll-like receptors, which bind to bacterial pathogen-associated molecular patterns, thereby activating nuclear factor kappa B (NFκB) signaling (Fournier, 2012). Cells can induce an inflammatory environment by secreting a series of cytokines and chemokines and utilize phagocytosis, reactive oxygen species (ROS), antimicrobial peptides, degranulation, and neutrophil extracellular traps to devour and kill bacteria (Amin Yavari et al., 2020). In addition, acquired immunity can be activated to produce antibodies that provide long-term protection against recurrent infections. In the absence of implants, planktonic bacteria can be eliminated by this immune response. In contrast, in IAI, the implant itself is recognized as a foreign body, thus activating the innate immune response. On the one hand, this leads to defects in the bacterial uptake and the killing ability of neutrophils, the underlying mechanisms of which include the over-generation of ROS, metabolic depletion, inflammasome activation, and even cell death. On the other hand, single macrophages become depressed due to their inability to engulf large foreign particles and fuse to form foreign body-responsive giant cells. Moreover, chronic infection causes macrophages to transform from the pro-inflammatory M1 to the anti-inflammatory M2 type, leading to a decline in sterilization capacity (Seebach and Kubatzky, 2019). The immune-compromised niche around the implant prevents bacteria from being effectively eliminated, eventually leading to the development of a persistent chronic infection (Orapiriyakul et al., 2018).

Bacteria can also disrupt bone homeostasis around the implants. In *S. aureus*-related IAI, *S. aureus* first binds to the bone ECM (type I collagen, fibronectin, and fibrinogen) through MSCRAMMs and adheres and aggregates around osteoblasts. With the help of the fibronectin bridge formed by fibronectin-binding protein and α5β1 integrin, bacteria can be internalized into osteoblasts (Fowler et al., 2000). Bacteria can then induce the necrosis and apoptosis of osteoblasts *via* virulence factors such as phenol-soluble modulins, which leads to a decrease in osteogenic activity. Infected bacteria can activate the immune response through a series of inflammatory factors and chemokines (IL-6, IL-12, CXCL2, and CXCL8) (Josse et al., 2015). Infected osteoblasts can also increase osteoclastogenesis by releasing receptor activator for the nuclear factor-κB ligand and colony-stimulating factors. Reduced osteogenesis and increased osteoclast activity subsequently lead to bone resorption (Marriott, 2013).

### Host Cells and Implant Materials

Under normal circumstances, an ideal orthopedic biomaterial implanted in the body should have the following characteristics: (1) good biological and cytocompatibility; (2) adequate mechanical properties; (3) promotion of bone ingrowth and integration at the bone-host interface; (4) long-term antibacterial properties, especially those that prevent bacterial adhesion and inhibit biofilm formation; (5) good immunocompatibility; and (6) appropriate material degradation rate in the bone healing stage (in specific circumstances). The rapid integration of the biomaterial surfaces by host cells is a key factor in the success of the implant. In addition, according to the “race for surface” theory, the host cells and bacteria compete for the surface of the material (Subbiahdoss et al., 2009). The rapid integration of host cells within materials can prevent bacterial adhesion and colonization. On the other hand, when bacteria have the upper hand and adhere to the surface of the material in advance, this significantly affects the adhesion of host cells (Gristina, 1987). Moreover, host immunity cannot prevent bacterial colonization and biofilm formation (Stones and Krachler, 2016).

## Antibacterial Mechanisms of Antibacterial Metals and Alloys

### Antibacterial Metal Elements

Metals as antibacterial agents have long been used in daily life, agriculture, and medicine. In ancient times, containers made of copper (Cu) and silver (Ag) were used to store food and disinfect water. Cu salts were used to prevent mycosis of grapevines. Moreover, Cu and Ag have been used to prevent wound infections. In the post-antibiotic era, although the specific antibacterial mechanism of metals remains unclear, metals have been extensively investigated due to their excellent broad-spectrum antibacterial effects, multiple sterilization mechanisms, and lack of resistance. The antibacterial ability of metals is mainly determined by donor atom selectivity, reduction potential, and speciation (Lemire et al., 2013). Coordination chemistry and hard-soft acid base theory are the cornerstones to understand the selective bonding of metals and ligand atoms (Nies, 2003; Haas and Franz, 2009), which refers to the ability of metals to participate in reduction reactions (Harrison et al., 2007). Speciation means that metals are often present in the cell in the form of specific structural or functional proteins, rather than in a free form (Gadd, 2010). Metal-induced bacterial damage is generally considered as the result of cell membrane damage, protein-function inactivation, and oxidative stress. The most common antibacterial elements are Ag, Cu, and zinc (Zn), and others such as gallium (Ga), gold (Au), tin (Sn), and strontium (Sr) have certain antibacterial properties.

#### Ag

Ag has been used as an antibacterial agent for thousands of years. Because of its excellent antibacterial and anti-biofilm formation capabilities, Ag is currently the most studied metal and is used in various coatings, dressings, and alloy applications (Geng et al., 2017; Shivaram et al., 2017; Surmeneva et al., 2017). Several types of silver coatings for arthroplasty components exist in clinical practice, such as the Kyocera (Kyocera Group, Osaka, Japan) coating, Mutars components (Implantcast, Buxtehude, Germany), PorAg-coating, and others (Diez-Escudero and Hailer, 2021). Many observational studies found that silver coatings of arthroplasty components seemed to reduce the risk of the development and relapse of PJI in high-risk tumor patients (Fiore et al., 2021). However, no high-level evidence to support the use of silver coatings in orthopedics exists (Medellin et al., 2019). The generation and release of Ag ions is the most critical determinant of antibacterial ability, regardless of the form (i.e., metallic, ionic, or nanoparticle) (Chernousova and Epple, 2013). The Ag-containing metal surface has no antibacterial ability in an oxygen-free and dry environment, whereas it exhibits a certain antibacterial ability following its slow oxidization in air (Ferreri et al., 2015). The main mechanism of its antibacterial activity remains controversial. Generally, the antibacterial mechanism of Ag ions (Kedziora et al., 2018) involves three processes: (1) generation of ROS, which are highly active and unstable toxic oxygen molecules, including superoxide anion, peroxygen, and hydroxyl radicals, that can induce oxidative stress and apoptosis; (2) interaction with the cell envelope, leading to the separation of the outer bacterial cell membrane, destruction of its integrity, increased cell membrane permeability, and decreased ATP synthesis; and (3) combination with bacterial cell contents, including proteins, ribosomes, enzymes, and DNA, to inactivate functional proteins, denature enzymes, and promote DNA damage. The antibacterial ability of Ag ions is stronger than that of Cu and Zn ions, and Ag ions can exert strong bactericidal effects even at very low concentrations. The minimum inhibitory concentration (MIC) of Ag ions for both *S. aureus* and *E. coli* is 10^−7^ mol/L (Ning et al., 2015). As for the cytotoxicity, the median inhibitory concentration (IC_50_) values of Ag ions for L929 fibroblasts and MC3T3-E1 osteoblastic cells are 4.25 × 10^−6^ and 2.77 × 10^−6^ mol/L, respectively (Yamamoto et al., 1998). *In vitro* studies have shown that 2.5 × 10^−7^ mol/L to 10^−6^ mol/L of Ag^+^ exhibits antibacterial effects against *S. aureus* and *E. coli* (antibacterial rate > 90%) and no cytotoxicity against eukaryotic cells (Ning et al., 2015).

#### Cu

Cu has a killing effect on various bacteria, including those that are gram-positive (*S. aureus* and *S. epidermidis*), gram-negative (*E. coli* and *P. aeruginosa*), and multi-drug resistant (MRSA and MRSE), and viruses. The MIC of Cu^2+^ for *S. aureus* is in the range of 1–111 × 10^−6^ mol/L, whereas the IC_50_ values of Cu^2+^ for L929 fibroblasts and MC3T3-E1 osteoblastic cells are 4.15 × 10^−5^ and 1.59 × 10^−5^ mol/L, respectively (Yamamoto et al., 1998; Ning et al., 2015). Owing to its potent and rapid antibacterial properties, Cu is the first metal antibacterial agent approved by the US Environmental Protection Agency. The antibacterial ability of Cu largely depends on its form (ion or nanoparticle), oxidation state (Cu^0^, Cu^1+^, or Cu^2+^), and concentration. In addition, the contact distance between microorganisms and Cu-containing surfaces, application form (dry or wet), and ambient temperature affect its antibacterial effect (Mitra et al., 2020). Regardless of its form, the main mechanisms of Cu’s antibacterial effect include cell membrane damage, ROS generation, and contact killing, which is the most characteristic phenomenon of contact with bacteria on Cu-containing surfaces. Bacteria can be killed in a short time upon close contact with Cu-containing surfaces. Particularly, it takes a few minutes in a dry environment and several hours in a wet environment (Espirito Santo et al., 2011). Although the mechanisms involved in contact killing are not well-understood, current evidence shows that a Cu-containing surface first releases Cu ions, which bind to the negatively charged peptidoglycans of the outer bacterial cell membrane, resulting in damage to the outer cell membrane, leakage of cell contents, and the subsequent inhibition of the intracellular respiratory chain activity to generate ROS, cause oxidative stress damage, and lead to apoptosis (Grass et al., 2011). A recent study on Cu-containing stainless steel found that contact killing may be mediated by electron transfer caused by the potential difference between the stainless steel and the Cu-rich phase on the surface, which in turn leads to proton consumption, inhibition of ATP synthesis, and generation of ROS, eventually leading to cell damage (Zhang et al., 2020c). Reducing the surface roughness can enhance this close and continuous contact-killing effect and improve sterilization efficiency (Zhang et al., 2021b).

Cu is also an indispensable trace element for life and participates in various physiological and biological functions in humans as ions. Most Cu in the body is distributed in the bones and muscles, and that ingested from food is mainly excreted through the bile. Maintaining Cu homeostasis is important because both its deficiency and excess can cause many diseases (Wang et al., 2021). The minimum intake recommended by the US Institute of Medicine to prevent Cu deficiency in adults is 0.9 mg/day, with a tolerable upper limit of 10 mg/d (Trumbo et al., 2001). In recent years, other biological effects of Cu, such as in osteogenesis and angiogenesis, have been revealed, increasing its potential applications as implants in orthopedics and cardiovascular systems (Jacobs et al., 2020). In summary, Cu can be added to existing alloy systems as an antibacterial element to endow the alloy with antibacterial properties.

#### Zn

Zn is a transition metal element in the fourth cycle and an essential element in the human body. It directly participates in the synthesis of enzymes and promotes the growth and development of the body, as well as tissue regeneration (Bai et al., 2020). The concentration of Zn ions is a key factor in bacterial growth: low concentrations of Zn can promote the growth of bacteria, whereas excessive Zn can produce bactericidal effects. The MIC and minimum bactericidal concentration of Zn^2+^ for *S. aureus* are 10^−7^ mol/L and 2.5 × 10^−7^ mol/L, respectively (Ning et al., 2015). The IC_50_ values of Zn^2+^ for L929 and MC3T3-E1 are 9.28 × 10^−5^ and 9 × 10^−5^ mol/L, respectively, which are higher than those of Ag and Cu ions (Yamamoto et al., 1998). The main antibacterial mechanism of Zn^2+^ is the destruction of the outer membrane of the bacteria, its binding to enzymatic active sites, and competitive inhibition of the biological activity of the cell (Ye et al., 2020). Compared with Zn^2+^, ZnO has a stronger antibacterial ability, especially at the nanometer level. Various coatings loaded with ZnO nanoparticles have been designed to treat IAI and fungal infections (Sirelkhatim et al., 2015; Araujo et al., 2020; Sanchez-Lopez et al., 2020). Compared with Zn^2+^, ZnO nanoparticles are more likely to destroy bacterial membranes, induce oxidative stress, and generate ROS, which in turn kill bacteria.

#### Ga

Ga has bactericidal activity against various pathogens, including multidrug-resistant bacteria (Minandri et al., 2014). Ga^3+^ and Fe^3+^ have similar ionic radii, potential energies, and electron affinities. Thus, exogenous Ga can replace Fe and bind to necessary proteins and enzymes to disrupt iron metabolism and change the living environment of bacteria, thus exerting antibacterial effects (Kaneko et al., 2007). Besides its antibacterial effects, Ga was reported to have a fungistatic effect against several fungi, such as *Candida* species. An *in vitro* study found that this effect may be attributed to iron disruption because Ga acted as a “Trojan horse” inside the fungi, just like its mechanism in bacteria (Bastos et al., 2019).

#### Au

Au is a chemically inactive metal. By virtue of its low reducibility, Au is unlikely to be oxidized by dissolved oxygen. Therefore, Au ions and nanoparticles have minimal ion release in biological fluids (Godoy-Gallardo et al., 2021). Many studies have demonstrated the antibacterial properties of Au (Zheng et al., 2017; Wang et al., 2020). Au mainly exerts antibacterial effects in the form of nanoparticles or nanoclusters. Compared with nanoparticles of other metals such as Ag, Cu, and Zn, Au nanoparticles have higher biological safety and relatively weak antibacterial ability. Possible mechanisms include direct contact with the outer membrane of bacteria causing membrane disruption, the production of ROS, photothermal effects, and photocatalysis (Liu et al., 2020). Among them, the membrane damage caused by direct contact plays a major role (Liu et al., 2020). Au is mainly used as a coating in tooth restoration, and there is no report on the application of Au alloys in orthopedics.

### Antibacterial Mechanisms of Alloys

Doping antibacterial metal elements into existing materials, antibacterial metals, and alloys is conducted through appropriate processing technology. To retain its original properties, the alloy is also imparted with continuous antibacterial properties, which is a novel method to prevent and treat IAI. The specific mechanism of the antibacterial action of alloys is complicated (Zhang et al., 2021a), and to date, four potential antibacterial mechanisms have been proposed. First, when the intermetallic phase precipitated on the alloy surface comes into contact with bacteria, contact killing can destroy the outer bacterial membrane, prevent bacterial adhesion, and inhibit biofilm formation. Second, the alloy can continuously release an appropriate amount of metal ions during corrosion or degradation to kill bacterial cells. Third, the environment around the implant undergoes a change in pH. For example, upon the degradation of a magnesium (Mg) alloy, an alkaline environment can form and is not conducive to bacterial growth. Lastly, the potential difference between the components in the alloy causes electron transfer, consumes H^+^, affects the activity of the proton pump, and releases a large amount of ROS, which in turn produces a killing effect. Overall, the antibacterial effect of alloys depends on, but is not limited to, various factors such as the incorporated elements, dosage, designed alloy system, body fluids, and the bacteria tested. Moreover, the addition of alloying elements inevitably affects the other properties of the material, such as its mechanical characteristics and corrosion behavior. Fortunately, advanced process technology minimizes the impact of other properties of the material to impart long-term antibacterial properties, thereby widening their application in the prevention and treatment of IAI.

## Applications of Common Antibacterial Metals and Alloys in IAI

### Antibacterial Ti Alloy

Due to its excellent biocompatibility, superior corrosion resistance, light weight, high tensile strength, and magnetic resonance imaging compatibility, Ti is widely used in orthopedic and dental implant materials, including artificial joint prostheses and fracture fixation plates, nails, rods, screws, wires, and dental and cardiovascular implants (Kaur and Singh, 2019). Currently, the most widely used alloys are commercially pure Ti (grade 4) and Ti6Al4V alloys. However, the main defects of Ti alloys are poor wear resistance, low fatigue strength, and relatively expensive cost. The main clinical concerns include IAI and stress shielding caused by high elastic modulus, which leads to bone loss and implant loosening. In addition, because very small amounts of vanadium and aluminum ions are precipitated by Ti6Al4V alloys, this can cause reduced cell adaptability and may harm the human body. The design of new Ti alloys is a research hotspot, and other related studies on Ti-Nb-Ta-Zr alloys, Ti-Mo alloys, and Ti-Al-Sr-Zr alloys are currently underway (Niinomi et al., 2012). To address the abovementioned problems, antibacterial elements such as Cu, Ag, and Ga are added to the alloy to improve its performance. The generated antibacterial Ti alloy has undoubtedly revolutionary and promising applications as medical implants in orthopedics and dentistry, especially for preventing IAI. **Table 1** summarizes the recent developments on the design of antibacterial Ti alloys.

**Table 1:** Engineered titanium alloys with antibacterial properties.

Alloy system	Sample	Preparation	Bacterial Strains	Antibacterial Test	Antibacterial Effect	Antibacterial Mechanisms		Application	Reference
Ti-Cu	Ti-xCu (x = 1 and 5 wt%)	/	*S. aureus, E. coli*	Plate counting	Evidently inhibited bacteria colonization	Cu-ion release	Prevention of pin tract infection		Shirai et al., 2009
				Rabbit pin tract infection model	Ti-1Cu alloy significantly inhibited inflammation and infection				
Ti-Cu	Ti-10 wt% Cu	Powder metallurgy	*S. aureus, E. coli*	Plate counting	Antibacterial rates for *E. coli* and *S. aureus*: 99%, 100%, respectively	Cu-ion release	Dental materials and surgical implant		Zhang et al., 2013
Ti-Cu	Ti–xCu (x = 2, 5, 10 and 25 wt%)	Powder metallurgy	*S. aureus, E. coli*	Plate counting	Cu content must be at least 5 wt% to obtain strong and stable antibacterial property	Cu-rich phase	Orthopedic and prosthodontic fields		Liu et al., 2014
Ti-Cu	Ti-xCu (x = 5 and 10 wt%)	Sintering	*P. gingivalis*	Plate counting, live/dead staining, and SEM	Killed anaerobic bacteria and reduced the activity of surviving bacteria	Cu ions released from Ti-Cu alloy	Dental implants		Bai et al., 2016
Ti-Cu	Ti-10Cu	Sintering	*S. aureus*	Infected rabbit muscle model	Reduced implant-related infection or inflammation	/	Orthopedic surgery and dental implant		Wang et al., 2019
Ti-Cu	Ti-3Cu	Microwave sintering	*S. aureus, E. coli*	Plate counting	Strong antibacterial ability and comparable elastic modulus with cortical bone	/	Orthopedic and dental implants		Tao et al., 2020
Ti–Cu	Ti-xCu (x = 2, 3 and 4 wt%)	Casting with post-treatment	*S. aureus*	Plate counting	Heat treatment significantly improved antibacterial rate	Homogeneous distribution and a fine Ti_2_Cu phase	Load-bearing implants and dental implants		Zhang et al., 2016a
Ti-Cu	Ti-5wt% Cu	Casting	*S. mutans, P. gingivalis*	Real-time PCR, live/dead staining, SEM, TEM	Antimicrobial/anti-biofilm activities	Cu ions released from the alloys	Dental implant		Liu et al., 2016b
Ti-Cu	Ti-5wt% Cu	Casting with post-treatment	*S. aureus, E. coli*	Plate counting, live/dead staining, SEM, TEM	Killed attached bacteria and inhibited biofilm formation	Contact sterilization	Dental application		Liu et al., 2018
				Dog mandibular premolar infection model	Superior capacities in inhibiting bone resorption				Liu et al., 2018
Ti6Al4V-Cu	Ti6Al4V-xCu	Casting with post-treatment	*S. aureus, E. coli*	Plate counting	Strong antibacterial abilities	/	Surgical implant materials		Ren et al., 2014
Ti6Al4V-Cu	Ti6Al4V-5Cu	Casting with post-treatment	*S. aureus*	Plate counting, SEM, live/dead staining	Annealing Ti6Al4V-5Cu alloy at 740°C showed the best overall properties	Ti_2_Cu phases	Bone implant		Peng et al., 2018
Ti6Al4V-Cu	Ti6Al4V-6.5wt%Cu	As-cast	*S. aureus*	Live/dead staining	Significant antibacterial effects and inhibited biofilm formation	/	Bone implant		Yang et al., 2021
Ti6Al5V-Cu	Ti6Al-4V-5.56 wt%Cu	As-cast	*MRSA*	Plate count method, crystal violet staining, SEM, and real-time PCR	Effectively killed MRSA and inhibited biofilm formation	Continuous and stable Cu^2+^ release	Implant material for protection against MRSA-induced IAI		Zhuang et al., 2021
			*MRSA*	Rat implant-associated infection model	No sign of infection				Zhuang et al., 2021
Ti6Al4V-Cu	Ti6Al4V-xCu (x = 0, 2, 4, 6 wt%) alloy	SLM	*S. aureus, E. coli*	Plate counting	Alloys with 4 wt% and 6 wt% Cu had strong and stable antibacterial properties	Cu ions release	Dental implant		Guo et al., 2017
Ti5Al2.5Fe-Cu	Ti5Al2.5Fe-xCu (x = 1, 3 and 5 wt%)	Powder metallurgy	*S. aureus, E. coli*	Plate counting	Antibacterial ability was enhanced by addition of Cu to Ti–5Al–2.5Fe alloy	Ti–Cu phases	Orthopedic and dental implants		Yamanoglu et al., 2018
Ti-Cu-Mn	Ti-xCu-yMn	Powder metallurgy	*E. coli*	Plate counting	Strong antibacterial activity	Ti_2_Cu intermetallic particles	Dental and orthopedic implants		Bolzoni et al., 2020
Ti-Nb-Ta-Zr-Cu	Ti-1.6Nb-10Ta-1.7Zr-xCu (x = 1, 3, 5, 10, and 11 wt%)	Casting with appropriate heat treatment	*S. epidermidis*	Bacterial luminescence	Good antibacterial effect	Larger amounts of Ti_2_Cu	Dental implants		Fowler et al., 2019
Ti-Ag	Ti–xAg (x = 1, 3 and 5 wt%)	Sintering	*S. aureus*	Plate counting	Ag content should be >3 wt% to achieve strong and stable antibacterial activity	Ti_2_Ag and its distribution	Orthopedic and dental implants		Chen et al., 2016
Ti-Ag	Ti-3Ag (sintered); Ti-3, 15Ag (T4); Ti-3, 15 Ag (T6)	Sintering, casting, casting with appropriate post-treatment	*S. aureus*	Plate counting	T6 treatment provided alloy with strong antibacterial ability	Ag ion release and homogeneously distributed Ti_2_Ag particles (key)	Orthopedic and dental implants		Chen et al., 2017
Ti-Ag	Ti-7, 9, 11 wt% Ag (T6); Ti-7, 9, 11 wt% Ag (ST);	Casting with appropriate post-treatment +/- surface treatment (ST)	*S. aureus*	Plate counting	Antibacterial properties increased with increasing Ag content	Ti_2_Ag particles in a contact sterilization mode (key); Ag ion release	Orthopedic and dental implants		Shi et al., 2020
Ti-Ag	Ti-xAg (x = 0, 1, 3 and 5 wt%)	Spark plasma sintering and acid etching	*S. aureus*	Plate counting	Antibacterial rates of Ti-1, 3, 5 wt% Ag were <21%	Particles with high Ag contents	Orthopedic and dental implants		Lei et al., 2018
Ti-Ga	Ti-8Al-3Si-3Zr-x1Ga (x = 1, 2 and 20 wt%); Ti-23Ga	Powder metallurgy	Multidrug-resistant *S. aureus*	Plate counting	Metabolic activity reduced by >80%	Surface-exposed Ga	Orthopedic applications		Cochis et al., 2019

“/” means “not mentioned”.

Copper-containing Ti alloys, including Ti-Cu, Ti6Al4V-Cu, Ti-Ni-Cu, Ti5Al2.5Fe-Cu, and Ti-Nb-Ta-Zr-Cu, have been widely investigated. For instance, in 2009, Shirai et al. reported that Ti-Cu alloys have antibacterial effects and can be used to prevent orthopedic external fixation nail tract infections. Subsequently, a variety of methods, including conventional powder metallurgy with vacuum protection, casting, spark plasma sintering, additive manufacturing, and microwave sintering have been used to prepare Cu-containing Ti alloys. In 2013, a Ti-10 wt% Cu sintered alloy was developed by hot pressure sintering, and its antibacterial rates against *S. aureus* and *E. coli* were 100% and 99%, respectively (Zhang et al., 2013). In addition, as long as the Cu content in the Ti-Cu sintered alloy exceeds 5 wt%, a continuous and stable antibacterial effect can be obtained (Liu et al., 2014). It was effective against not only *S. aureus* and *E. coli* but also the common anaerobic bacterium *Porphyromonas gingivalis* (Bai et al., 2016). The excellent antibacterial effects and good biocompatibility of the Ti-10Cu sintered alloy have been verified in a rabbit muscle infection model (Wang et al., 2019). Recently, a Ti-3Cu alloy with a porous structure was prepared by microwave sintering (Tao et al., 2020). Although its Cu content is lower than the 5% required by the traditional metallurgical method, *in vitro* studies have shown that its antibacterial rate against *S. aureus* and *E. coli* can reach 100% after 12 h of incubation. Moreover, its elastic modulus was significantly lower than that of pure Ti and was remarkably close to that of human cortical bone, thus preventing prosthesis loosening caused by stress shielding (Tao et al., 2020).

Unlike the powder metallurgy method, which uses metal powder for compression molding, sintering, and post-processing, casting is a processing method in which solid metal is melted into a liquid, poured into a specific mold, solidified, and formed. Zhang et al. used casting and heat treatment methods to prepare Ti-Cu alloys with Cu contents of 2%, 3%, and 4%. They found that pure as-cast Ti-Cu alloy exhibited extremely low resistance to *S. aureus* adhesion, whereas heat treatment, especially after solid solution and aging, significantly improved antibacterial rate. The antibacterial rates of the Ti-3Cu and Ti-4Cu alloys were 90.33% and 92.57%, respectively. Furthermore, mechanical strength and corrosion resistance were significantly enhanced. These performance optimizations are attributed to the uniformly distributed Ti_2_Cu phase (Zhang et al., 2016a). In addition, following the proper heat treatment of as-cast Ti-Cu alloy, an alloy whose copper content is >3 wt% can obtain good antibacterial properties and satisfactory mechanical and corrosion resistance (Zhang et al., 2016a). *In vitro* studies have shown that as-cast Ti-5Cu alloy not only effectively kills attached bacteria (*P. gingivalis* and *S. mutans*), but also significantly inhibits biofilm formation (Liu et al., 2016b). After 24 h of incubation, the number of viable bacteria in the biofilm on the Ti-Cu alloy surface was significantly less than that on the pure Ti surface. The surface of pure Ti had *S. mutans* biofilm (40 μm), whereas that of Ti-Cu had no biofilm. Meanwhile, the biofilm of *P. gingivalis* on the surface of Ti-Cu was only half as thick as that of pure Ti. In addition, according to TEM images, the peptidoglycan layer of the bacteria in contact with the Ti-Cu alloy surface was degraded, the plasmonic wall was separated, and the contents showed leakage. In contrast, the bacterial cell membrane in contract with the pure Ti surface was intact and regularly shaped. These results confirm the contact-killing mechanism of the Ti-Cu alloy (Liu et al., 2016b). In subsequent studies, the same research team found that Ti-5Cu alloy exerts clear anti-adhesion and anti-biofilm properties against *S. aureus* and *E. coli in vitro*. Using an *in vivo* peri-implantitis model, they further showed that Ti-Cu alloy is superior to pure Ti alloy in inhibiting osteolysis caused by bacterial infection and promoting bone formation (Liu et al., 2018).

In 2014, Ren et al. first reported the antibacterial properties of Ti6Al4V alloys containing Cu (Ren et al., 2014). The Ti6Al4V-xCu alloy (x = 1, 3, and 5 wt%) prepared by casting and proper heat treatment has unique antibacterial effects, corrosion resistance, and biocompatibility. As the copper content increases, the inhibition effect against the growth of *S. aureus* and *E. coli* also increases. Different annealing temperatures during heat treatment have a significant impact on the antibacterial and anti-biofilm activities of the Ti6Al4V-5Cu alloy (Peng et al., 2018). Upon treatment at a relatively low temperature (740°C), the alloy showed stronger antibacterial and anti-biofilm capabilities than that at relatively high temperatures (820–910°C). Moreover, this low temperature-treated Ti6Al4V-5Cu alloy showed the best mechanical properties, corrosion resistance, and biocompatibility. The authors attributed these results to the preferential adhere of bacteria to the α phase compared with the β phase or Ti_2_Cu region (Peng et al., 2018). Subsequently, they compared the time-effect relationship of biofilm formation by *S. aureus* on the surface of Ti6Al4V-6.5Cu and Ti6Al4V alloys and found that the former can slow down the adhesion of bacteria on the surface of the material, accelerate the aging of the biofilm, and inhibit biofilm formation (Yang et al., 2021). Zhuang et al. (2021) investigated the antibacterial performance of a Ti6Al4V-Cu alloy against MRSA using plate counting and found an antibacterial rate of 99.7% MRSA after 24 h of incubation. Its anti-biofilm formation activity was confirmed using crystal violet staining and scanning electron microscopy. Real-time PCR results showed that the expression of biofilm formation-related genes *icaA/B/D/C/E* in the Ti6Al4V-Cu group was downregulated. In addition, the levels of virulence-related (*fnbA*) and antibiotic resistance mRNA (*mecA*, *femA*, *femB*, and *femX*) were significantly lower in the Ti6Al4V-Cu group than in the Ti6Al4V group. They also established a rat IAI model with MRSA to evaluate the *in vivo* antibacterial effect of the Ti6Al4V-Cu alloy. Radiological evaluation revealed that at 3 weeks, the mice implanted with Ti6Al4V alloys developed typical IAI manifestations, including purulent exudate, osteolysis, and periosteal reaction, whereas those in the Ti6Al4V-Cu group showed only mild inflammation. At 6 weeks, the osteolysis reaction in the Ti6Al4V group was intensified, and dead bone was formed; however, there was no sign of infection in the Ti6Al4V-Cu group. The above results were further confirmed by bacterial cultures, histological analysis, and immunohistochemistry.

Selective laser melting is a novel 3D printing technology that uses metal powder to melt and rapidly solidify under the heat of a laser beam. Guo et al. prepared a Cu-bearing Ti6Al4V alloy using selective laser melting (SLM). *In vitro* results showed that Ti6Al4V-4Cu and Ti6Al4V-6Cu have strong and stable antibacterial effects against *S. aureus* and *E. coli* (Guo et al., 2017). Remarkably, Ti6Al4V-6Cu did not affect the viability of osteoblasts, inhibited the inflammatory response of macrophages, and promoted the formation of human vascular endothelial cells (Xu et al., 2018). These characteristics show good prospects for the prevention and treatment of orthopedic IAI. However, due to the potential toxicity of Al and V ions, Cu has been added to other Ti alloys such as Ti5Al2.5Fe, Ti-Nb-Ta-Zr, and Ti-Mn alloys to endow these alloys with antibacterial properties (Yamanoglu et al., 2018; Fowler et al., 2019; Bolzoni et al., 2020).

Ti-Ag alloys can also be prepared by traditional powder metallurgy, spark plasma sintering, and casting methods. For example, Chen et al. used Ti and Ag powders of different sizes to fabricate Ti-Ag alloys by traditional powder metallurgy (Chen et al., 2016). *In vitro* results showed that the antibacterial effect and corrosion resistance of Ti-Ag showed a dose-dependent relationship with Ag content. Ag content > 3 wt% achieved strong and stable antibacterial properties. This effect is attributed to the formation and distribution of the Ti_2_Ag phase. Subsequently, Chen et al. prepared Ti-Ag alloys by casting and heat treatment. Pure as-cast alloys exhibited a low antibacterial rate (<90%) against *S. aureus*, whereas the as-cast Ti-Ag alloy with solid solution and aging treatment had an antibacterial rate equivalent to that of the sintered Ti-3Ag alloy (>90%). Microstructure analysis revealed that after proper heat treatment, the as-cast Ti-Ag alloy had an evenly distributed nano-scale Ti_2_Ag phase, whereas the Ti-3Ag alloy had a rough micro-scale Ti_2_Ag layer. The nano-scale and micro-scale Ti_2_Ag layers both play a key role in the antibacterial process (Chen et al., 2017). Further research found that the main antibacterial mechanism of Ti-Ag alloy involves a contact-killing effect caused by the Ti_2_Ag particles. The Ag ions released also exert a minor effect. Surface treatment causes more Ti_2_Ag particles to exist on the surface of the alloy, thus increasing bacterial contact and improving the antibacterial effect (Shi et al., 2020). A new Ti-Ag alloy was successfully prepared by spark plasma sintering; however, its antibacterial rate was worse than that of the as-sintered Ti-Ag alloy by traditional powder metallurgy because there were fewer Ti_2_Ag ions on the alloy surface. However, the antibacterial rate can be significantly improved by acid-etching treatment without affecting biocompatibility (Lei et al., 2018). To date, Ti-Ag alloys have not been tested in *in vivo* studies; thus, their *in vivo* compatibility and effectiveness need further verification.

Cochis et al. formulated a Ga-containing Ti alloy by powder metallurgy. By adding different amounts of metallic Ga to the Ti alloy, they showed that its strong killing effect on multi-drug resistant *S. aureus* is time- and dose-dependent. Furthermore, the alloy can significantly reduce the number and vitality of biofilms. However, Ga^3+^ was not detected in the solution. They speculate that Ga exposed on the alloy surface is in direct contact with solvent-exposed [4Fe-4S] protein clusters, resulting in the toxic release of additional Fenton-active Fe into the cytoplasm to generate large amounts of ROS (Cochis et al., 2019).

### Antibacterial Co-Based Alloy

Co-based alloys are non-degradable materials commonly used in clinical practice, mostly as hip joint prostheses and dental restoration materials (Hedberg et al., 2015). Their most prominent features include good corrosion and wear resistance. However, they are associated with potential toxicity due to the released metal particles (Bradberry et al., 2014). Similarly, there is also the risk of IAI. The recent developments on antibacterial Co-based alloys are summarized in **Table 2**.

**Table 2:** Engineered cobalt-based alloys with antibacterial properties.

Alloy system	Sample	Preparation	Bacterial strains	Antibacterial test	Antibacterial effect	Antibacterial mechanisms	Application	Reference
CoCrWNi-Cu	CoCrWNi-xCu (x = 2, 4 wt%)	Casting	*S. aureus, E. coli*	Plate counting, SEM	Exerted antibacterial properties and inhibited the formation of bacterial biofilms on its surface	Release of Cu ions	Orthopedic and dental implant	Wang et al., 2014
CoCrMo-Cu	Co-29Cr-6Mo-xCu (x = 1, 4 wt%)	Casting	*S. aureus*	Plate counting	Strong antibacterial abilities	Cu phases	Orthopedic and dental implant	Zhang and Liu, 2016
CoCrMo-Cu	Co-29Cr-6Mo-xCu (x = 0.8, 1.8 and 3.6 wt.%)	Casting	*S. aureus*	Plate counting	Cu addition enhanced antibacterial properties	Cu phases	Orthopedic and dental implant	Li et al., 2019c
CoCrW-Cu	CoCrW-2.8 wt% Cu alloy	SLM	*S. aureus, E. coli*	Plate counting, live/dead cell staining	Bactericidal and inhibited biofilm formation	Cu ions released from its surface	Dental applications	Ren et al., 2016
CoCrW-Cu	CoCrWCu alloys with differing Cu content (2, 3, 4 wt%)	SLM	*E. coli*	Plate counting	Excellent antibacterial performance against *E. coli* when at least 3 wt% Cu	Cu ions released from its surface	Dental implant	Lu et al., 2018
CoCr-Ag	Co-30Cr-5Ag	Mechanical alloying and spark plasma sintering	*S. aureus, E. coli*	Plate counting, SEM	Ag addition significantly enhanced antibacterial activity (antibacterial rates: 90.5% and 72.6%, respectively)	Ag ions	/	Jiang et al., 2019

“/” means “not mentioned”.

Studies on antibacterial Co-based alloys mainly focused on Cu-containing Co-based alloys. Wang et al. fabricated a Cu-bearing CoCrWNi alloy by casting and used the plate-counting method to determine its antibacterial activity (Wang et al., 2014). They found that the CoCrWNi alloy containing 4.29 wt% Cu exerted >90% antibacterial rates against *S. aureus* and *E. coli*. SEM results confirmed that the CoCrWNi alloy containing 4.29 wt% significantly inhibits biofilm formation. This antibacterial ability was attributed to the released Cu^2+^ on the surface. In 2016, Zhang et al. successfully prepared a new antibacterial Co-29Cr-6Mo-Cu alloy by casting. The Cu content (1–4 wt%) was positively correlated with antibacterial performance; however, wear resistance was reduced. This was significantly improved after proper heat treatment (Zhang and Liu, 2016; Li et al., 2019c). Moreover, they found that Co-29Cr-6Mo-Cu, especially that with 2 wt% Cu, has the best osteogenic properties *in vivo*. They speculate that Cu^2+^ released from the Cu-rich phase promotes the expression of BMP2 and IGF-1 (Duan et al., 2020). Using the SLM method, Ren et al. successfully engineered a CoCrWCu alloy showing strong antibacterial and anti-biofilm properties (Ren et al., 2016). The 3 wt% CoCrWCu alloy is expected to become a dental restoration material candidate due to its superior corrosion resistance, mechanical properties, antibacterial properties, and biocompatibility (Lu et al., 2018). In addition to alloying Cu, a Co-30wt%Cr-5wt%Ag ternary alloy was developed by doping Ag into Co-Cr alloy and showed excellent antibacterial ability (Jiang et al., 2019). Although there are no *in vivo* studies on antibacterial Co-based alloys to date, they are promising materials for preventing IAI.

### Degradable Antibacterial Metals and Alloys

#### Antibacterial Mg Alloy

Degradable metals are a type of medical metal that are gradually degraded by body fluids after assisting the body in tissue repair. The corrosion products released elicit appropriate host responses. Medical-degradable alloys mainly include those based on Mg and Zn. Mg-based alloys were used because of their excellent physical and chemical properties and good biocompatibilities. The mechanical strength and ductility of Mg are better than those of Zn, and the elastic modulus (40–45 MPa) of Mg alloys is similar to that of natural cortical bone (10–30 MPa) (Kamrani and Fleck, 2019). Mg can also be relatively quickly degraded in the body, and its products have osteopromotive properties. Therefore, Mg-based materials can be used as suitable bone substitutes to provide initial support for bone injuries and fractures (Zhang et al., 2016b). In fact, commercial Mg-based alloys, such as MgYReZr degradable interface nails and screws made of Mg-Ca-Sr alloy, have been successfully approved for orthopedic surgery (Wang et al., 2020).

The main disadvantage of Mg is its rapid degradation rate in the body (generally 2–3 months). Mg alloy is also oxidized upon coming into contact with a liquid solution to produce Mg(OH)_2_ and H_2_. In a physiological environment, the hydroxide protective film on the surface of the precipitated alloy combines with chlorine to form soluble MgCl_2_, which increases the local pH. The local alkaline microenvironment corrodes the alloy until it is completely degraded (Luque-Agudo et al., 2020). Mg is suggested to exhibit good antibacterial properties *in vitro*, mainly due to the alkaline microenvironment formed by Mg degradation and the release of Mg^2+^ (Rahim et al., 2015). However, studies have revealed that the *in vivo* antibacterial properties of pure Mg and Mg alloys are significantly weakened and even aggravate the formation of biofilms. Possible causes include the buffering effect of body fluids resulting in insignificant pH changes, protein adsorption on the surface, and the hydrogen interface, which is caused by rapid degradation of pure Mg and Mg alloys (Rahim et al., 2016; Brooks et al., 2018; Rahim et al., 2020). To date, pure Mg and alloys have been modified by various methods, such as alloying and surface modifications, to enhance their antibacterial performance and improve corrosion resistance (Lin et al., 2021). Here, we enumerate the antibacterial Mg alloys prepared by alloying methods. There are four categories according to the alloying elements: Mg-Cu, Mg-Ag, Mg-Zn, and Mg-Ga (**Table 3**).

**Table 3:** Engineered magnesium alloys with antibacterial properties.

Alloy system	Sample	Preparation	Bacterial strains	Antibacterial test	Antibacterial effect	Antibacterial mechanisms	Application	Reference
Mg-Cu	Mg-xCu (x = 0.03, 0.19, and 0.57 wt%)	Casting	*S. aureus*	Plate counting	Enhanced long-lasting antibacterial effects	Mg_2_Cu intermetallic phases accelerated degradation and formation of the alkaline environment, along with Cu release	Orthopedic applications	Liu et al., 2016a
Mg-Cu	Mg-xCu (x = 0.05, 0.1 and 0.25 wt%)	Casting	*E. coli, S. epidermidis, MRSA*	Plate counting, bacterial viability assays, SEM, and PCR	Mg-0.25Cu exhibited excellent antibacterial performance	Cu-ion release	Treatment of orthopedic infections	Li et al., 2016
	Mg-0.25Cu		MRSA	Rabbit tibia osteomyelitis model	Effectively treated chronic osteomyelitis infection			Li et al., 2016
Mg-Cu	Mg-xCu (x = 0.1, 0.2 and 0.3 wt%)	Casting and extrusion with solution treatment	*S. aureus*	Plate counting	Reduced viability of *S. aureus*	High alkalinity and Cu-ion release	Treatment of IAI	Yan et al., 2018
Mg-Al-Cu	Mg-Al-xCu (x = 0, 0.25, 0.5 and 1 wt%)	Two-step mechanical alloying and spark plasma sintering	*S. aureus, E. coli*	Disc diffusion	Prevented bacterial growth according to the Cu content	Cu-ion release	Orthopedic implant	Safari et al., 2019
Mg-Ag	Mg-xAg (x = 6, 8 wt%)	Casting followed by a solidification cooling process	*S. aureus, S. epidermidis*	Live/dead staining	Killing rate exceeded 90%	Ag^+^ release	Orthopedic implant	Tie et al., 2013
Mg-Ag	Mg-xAg (x = 6, 8 wt%)	Casting followed by homogenization treatment and hot extrusion	*S. aureus, S. epidermidis*	Live/dead staining, CLSM	Good antibacterial properties by increasing the silver content	Ag^+^ release	Bone implant	Liu et al., 2017
Mg-Zn-Y-Nd-Ag	Mg-Zn-Y-Nd-xAg (x = 0.2, 0.4, 0.6, and 0.8 wt%)	Extrusion at 320°C	*S. aureus, E. coli*	Plate counting	Alloy containing 0.4 wt% Ag exhibited better antimicrobial properties and mechanical property	Ag ions	Treat orthopedic infections	Feng et al., 2018
Mg-Ca-Sr-Zn	Mg–1Ca–0.5Sr–xZn (x = 0, 2, 4, 6) alloys	Extrusion at 320°C	*S. aureus*	Plate counting, live/dead staining, SEM	Mg-Ca-Sr-6Zn alloy exhibited strong antibacterial effect	Combination of Zn^2+^ and Sr^2+^, rapid release of hydrogen gas and OH^-^	Antibacterial, biodegradable orthopedic implant	He et al., 2015
Mg-Zn-Ca	Mg-2Zn-0.5Ca	Melting, casting, extrusion, and drawing	MRSA	Plate counting, SEM	Reduced bacterial adhesion on the surface	Higher pH values and Mg-ion concentrations	Bone repair	Zhang et al., 2020a
Mg-Nd-Zn-Zr	Mg-Nd-Zn-Zr	Semi-continuous casting	*E. coli, S. aureus, S. epidermidis*	Spread plate, confocal CLSM, and SEM	Enhanced antibacterial activity	Zn and Zr on its surface, released Zn ions, and increased alkalinity with its degradation	Orthopedic implants	Qin et al., 2015
	Mg-Nd-Zn-Zr, Mg		*S. aureus*	Implant-related osteomyelitis model in rat femur	Mg and Mg-Nd-Zn-Zr reduced the risk of implant-related infections, and the latter had a better effect			Qin et al., 2015
Mg-Sr-Ga	Mg-0.1Sr, Mg-0.1Ga and Mg-0.1Sr-0.1Ga	Casting	*S. aureus, E. coli, S. epidermidis*	Spread plate, live/dead staining, CLSM	Mg-Sr-Ga alloys had the strongest germ-killing ability	Presence of Ga^3+^ and Sr^2+^	IAI	Gao et al., 2019
	Mg-0.1Sr, Mg-0.1Ga and Mg-0.1Sr-0.1Ga		*S. aureus*	*In vivo* implant-related osteomyelitis model in rat femur	Lowest number of *S. aureus* on the surface of the retrieved Ga-containing Mg rod implants			Gao et al., 2019

Mg-Cu alloys with specific Cu content exhibit osteogenesis, angiogenesis, and lasting antibacterial properties *in vitro* (Liu et al., 2016a). As-cast Mg-0.25 wt% Cu alloys can be used to treat MRSA-induced osteomyelitis in rabbits, and these effects have been confirmed by radiological images, microbiological cultures, and histopathological analysis (Li et al., 2016). The addition of Cu forms a Mg_2_Cu intermetallic phase, which accelerates the degradation of the Mg-Cu alloy and leads to the rapid release of Cu^2+^. In combination with the highly alkaline environment, the released Cu^2+^ ions are responsible for the antibacterial activity of Mg-Cu alloys. Accelerated corrosion can be controlled by reducing the copper content and using appropriate processing methods (Yan et al., 2018). In addition, Mg-Al-Cu alloys can reduce the degradation rate of Mg alloys while maintaining their antibacterial effects, mechanical properties, and biocompatibility, suggesting that Mg-Al-Cu alloy is a promising degradable material that can be used for bone implants (Safari et al., 2019).

The incorporation of Ag into Mg alloys can significantly improve corrosion resistance and enhance antibacterial performance. Tie et al. designed three Mg-Ag alloys with different Ag contents (2, 4, and 6 wt%) by casting and appropriate heat treatment (Tie et al., 2013). *In vitro* antibacterial assays carried out on a dynamic bioreactor system confirmed that their antibacterial rates against *S. aureus* and *S. epidermidis* are greater than 90% and that they are essentially non-toxic to osteoblasts. This antibacterial effect has been attributed to the microstructure of the alloy and the Ag content (Liu et al., 2017). Similarly, the addition of 4 wt% Ag to the extruded Mg-Zn-Y-Nd alloy showed good antibacterial activities, mechanical properties, and corrosion resistance (Feng et al., 2018).

The addition of Zn can also improve the properties of Mg alloys. For example, the Mg-Ca-Sr-Zn alloy significantly reduced the number of adherent bacteria; higher Zn content exerted a better antibacterial effect (He et al., 2015). The formation of oxides on the surface of the sample and the rapid generation of H_2_ may contribute to the observed antibacterial effect; the release of Zn and Sr ions may also enhance this effect. Another study reported that the antibacterial effect of Mg-Zn-Ca on MRSA is stronger than those of pure Mg and Mg-Zn-Sr alloys (Zhang et al., 2020a). In addition, adding an appropriate amount of Nd, Zn, and Zr to Mg alloys can significantly enhance corrosion resistance and antibacterial properties. In a rat femoral osteomyelitis model, the *in vivo* antibacterial effect of the Mg-Nd-Zn-Zr alloy was successfully verified, thus providing a new strategy for treating IAI (Qin et al., 2015).

Lastly, Gao et al. prepared Mg-0.1Sr-0.1Ga alloy by micro-alloying and observed its high killing effect on both planktonic and attached bacteria. The release of Ga^3+^ and Sr^2+^ ions played an essential role in this antibacterial function. In addition, no intermetallic phase was found in the alloy, indicating that the addition of Sr and Ga improves the corrosion resistance of Mg (Gao et al., 2019).

#### Antibacterial Zn Alloy

The degradation rate of Zn is slower than that of Mg as its standard electrode potential (−0.763 V) is higher than that of Mg (−2.37 V). Thus, Zn materials generally begin to degrade 4–6 months after implantation in the body (Mostaed et al., 2018). Hydrogen evolution does not occur during Zn degradation. Therefore, Zn alloys have become a research hotspot for medical biodegradable metals, especially in orthopedics, craniofacial and oral surgery implants, and cardiovascular stents (Hernandez-Escobar et al., 2019; Su et al., 2019). However, Zn has poor mechanical properties and ductility. Particularly, the tensile strength, yield strength, and elongation of as-cast pure Zn are 20 MPa, 30 MPa, and 1.2%, respectively, which are far below the minimum requirements for implants with load-bearing applications (tensile strength > 300 MPa, yield strength > 230 MPa, and elongation > 15–18%) (Kabir et al., 2021). Fortunately, metal alloying and several preparation techniques and post-processing methods can optimize the microstructure of Zn to enhance its mechanical properties (Venezuela and Dargusch, 2019). Alloying not only improves the mechanical properties of materials for load-bearing applications but also endows them with excellent antibacterial properties. The recent progress on antibacterial Zn alloys for implant applications is summarized in **Table 4**. The most common alloying elements are Cu, Ag, and Mg.

**Table 4:** Engineered zinc alloys with antibacterial properties.

Alloy system	Sample	Preparation	Bacterial strains	Antibacterial test	Antibacterial effect	Antibacterial mechanisms	Application	Reference
Zn-Cu	Zn-4.0 wt.%Cu	Extrusion at 280°C	*S. aureus*	Live/dead cell staining	Effectively inhibited bacteria adhesion and biofilm formation	Release of Cu and Zn ions	Vascular stents	Niu et al., 2016
Zn-Cu	Zn-xCu (x=1, 2, 3, and 4 wt%) alloys	Extrusion at 280°C	*S. aureus*	Inhibition zone diameter (IZD) test	Antibacterial property was perfect when Cu concentration > 2 wt%	Release of Cu and Zn ions	Cardiovascular implants	Tang et al., 2017
Zn-Cu	Zn-xCu (x = 1, 2 and 4 wt%) alloys	Casting followed by heating to 350°C for 1 h and rolling	Mixed oral bacteria	Live/dead cell staining	Zn-4Cu alloy can inhibit biofilm formation of mixed oral bacteria	Release of Zn^2+^ and Cu^2+^ and increased pH (OH^-^ release)	Osteosynthesis implants, especially in the craniomaxillofacial area	Li et al., 2019b
Zn-Cu-Fe	Zn-3Cu-xFe (x = 0, 0.2, 0.5 wt%)	Extrusion at 180°C	*S. aureus, E. coli*	Plate counting	Antibacterial properties of Zn-3Cu alloy were significantly improved by Fe alloying	Higher degradation rate and more Zn^2+^ and Cu^2+^ released	Vascular stents	Yue et al., 2020
Zn-Cu-Ti	Zn-1Cu-0.1Ti alloy	Casting and plastic deformation processes including hot-rolling and cold-rolling	*S. aureus*	IZD)	Good antibacterial effect with higher IZD than pure Zn	/	Bone fracture fixation applications such as bone screws and plates of biodegradable implants	Lin et al., 2020
Zn-Cu	Zn-xCu (x = 0, 0.5, 1 and 2 wt%)	extruding	*S. aureus, S. epidermidis, MRSA, MRSE*	Plate counting, live/dead staining, FESEM, TEM, real-time PCR of bacteria-related genes	Prevented bacterial adhesion and biofilm information	Inhibition of expression of genes related to wall synthesis, adhesion, colonization, biofilm formation, autolysis, and secretion of virulence factors in MRSA	Biodegradable orthopedic materials	Qu et al., 2020
	Zn-2Cu		MRSA	Rat femur intramedullary nail infection prevention model	Significant antibacterial activity against MRSA and reduction of inflammatory toxic side-effects and infection-related bone loss			Qu et al., 2020
Zn-Ag	Zn-4.0Ag alloy	Casting followed by thermomechanical treatment	*S. gordonii*	Crystal violet staining, live/dead cell staining	Effectively inhibited initial bacteria adhesion	Released Zn and Ag ions	Craniomaxillofacial osteosynthesis implants	Li et al., 2018
Zn-Ag-Au-V	Zn–2Ag–1.8Au–0.2V (wt.%) alloy	Casting followed by hot-rolling at 200°C and annealing at 390°C for 15 min	*S. gordonii*	Live/dead cell staining	Reduced plaque formation, inhibited bacterial adhesion and biofilm formation	Degradation products, such as released Zn^2+^, Ag^+^, and OH^−^	Craniomaxillofacial osteosynthesis implants	Li et al., 2019a
Zn-Mg	Zn-0.02 Mg alloy	Extrusion at 180°C	*S. aureus, E. coli*	Spread plate assay, live/dead viability assay, SEM	Strong antibacterial effect	Zn^2+^ damaged bacterial cell membranes and inhibited the multiple bacterial activities, such as nutrient transport and glycolysis	Cardiovascular stents	Lin et al., 2019
Zn-Al-Mg	Zn-0.5Al-xMg (x = 0, 0.1, 0.3 and 0.5 wt%)	Casting followed by suitable post-treatment	*E. coli*	Disc diffusion antibiotic sensitivity testing	Zn-0.5Al-0.5Mg significantly prohibited the growth of *E. coli*	/	Biodegradable orthopedic materials	Bakhsheshi-Rad et al., 2017
Zn-Mg-Sr	Zn-0.8Mg-0.2Sr (wt%)	Combination of casting, homogenization annealing, and extrusion at 200°C	*S. gordonii*	Live/dead staining	Effective inhibition of initial adhesion and biofilm formation	Released Zn^2+^ and alkaline shift in pH	Cranial and maxillofacial implants	Capek et al., 2021

“/” means “not mentioned”.

Niu et al. alloyed Zn with 4 wt% Cu using hot extrusion. *In vitro* tests showed its inhibition of bacterial adhesion and biofilm formation of *S. aureus*. This antibacterial effect is mainly due to the release of Zn^2+^ and Cu^2+^ (Niu et al., 2016). In addition, the effects of different Cu contents on the mechanical properties, corrosion behavior, and antibacterial properties of as-extruded Zn-Cu alloys were investigated. Increasing Cu content significantly improved the mechanical properties of the alloy, and perfect antibacterial effects were obtained at >2 wt% Cu (Tang et al., 2017). Binary Zn-Cu alloys were also prepared by rolling. The Zn-4 wt% Cu alloy had enhanced mechanical properties compared with the as-extruded Zn-Cu alloy and successfully inhibited the adhesion of oral mixed bacteria and biofilm formation, thus showing promising potential for craniomaxillofacial osteosynthesis implants (Li et al., 2019b). Yue et al. (2020) added antibacterial Cu and Fe to Zn-based materials to synthesize the Zn-3Cu-xFe (x = 0, 0.2, and 0.5 wt%) alloy. *In vitro* assay results showed that Zn-3Cu-0.2Fe and Zn-3Cu-0.5Fe alloys exhibited bacteriostatic rates of >90% against *S. aureus* and *E. coli*. Furthermore, increasing Fe content improved the low-concentration antibacterial effects of the alloy. This increased antibacterial ability was due to the action of Fe, which accelerated alloy degradation to increase free Zn^2+^ and Cu^2+^, which greatly enhanced antibacterial performance (Yue et al., 2020). Similarly, a Zn-1Cu-0.1Ti alloy exhibiting good antibacterial activity and mechanical properties was reported as a potent candidate for bone fracture fixation applications (Lin et al., 2020). However, its *in vitro* antibacterial effects were not fully reflected by the effects observed *in vivo*. Recently, Qu et al. (2020) investigated the *in vitro* and *in vivo* antibacterial effects of Zn-Cu alloy. *In vitro* antibacterial assays showed that the Zn-2Cu alloy had a clear killing effect on *S. aureus, S. epidermidis*, MRSA, and MRSE. Using a rat femur intramedullary nail infection prevention model, the Zn-2Cu alloy showed potent antibacterial activity against MRSA and reduced infection-related bone loss. To further explore its potential antibacterial mechanisms, real-time PCR was used to identify the genes related to MRSA-related behaviors (including adhesion, biofilm formation, autolysis, cell wall synthesis, drug resistance, and virulence). They showed that mice implanted with the Zn-2Cu alloy had significantly downregulated genes involved in biofilm formation (*icaA/B/C/D*), adhesion (*atlE*), cell wall synthesis, resistance, and virulence. This suggests that the Zn-2Cu alloy exhibits antibacterial behavior mainly by inhibiting cell wall synthesis, adhesion, biofilm formation, autolysis, and secretion of virulence factors. Moreover, the Zn-2Cu alloy has excellent mechanical properties and good biocompatibility. Therefore, it is a promising candidate for the prevention and treatment of IAI.

Adding Ag to Zn-based alloys can also enhance their mechanical properties and antibacterial activities. A binary Zn-4.0Ag alloy made by casting and proper treatment showed enhanced mechanical properties and effectively inhibited the adhesion and biofilm formation of *S. gordonii* (Li et al., 2018). Similarly, the quaternary Zn-2Ag-1.8Au-0.2V alloy exhibited an antibacterial effect (Li et al., 2019a).

Lastly, the addition of Mg to Zn-based alloys has also been explored to impart antibacterial properties. Lin et al. designed a binary Zn-Mg alloy and showed that Zn-0.02Mg had a strong *in vitro* antibacterial effect on *S. aureus* and *E. coli*. This is attributed to the action of Mg in accelerating corrosion, thereby increasing the quantity of released Zn^2+^ (Lin et al., 2019). A similar phenomenon was observed in a ternary Zn-Al-Mg alloy. Higher Mg content resulted in a stronger antibacterial effect on *E. coli* (Bakhsheshi-Rad et al., 2017). In addition, adding an appropriate amount of Sr to the Zn-Mg alloy significantly improved its mechanical properties and effectively inhibited the adhesion and biofilm formation of *S. gordonii in vitro* (Capek et al., 2021).

### Antibacterial Ta Metal and Alloy

Ta has excellent biological inertness, good biocompatibility, and strong corrosion resistance due to the dense Ta_2_O_5_ protective film on its surface. Ta has higher osseointegration capabilities than Ti (Han et al., 2019). Owing to its excellent mechanical properties, osteoinduction, bone conduction, and osseointegration, porous Ta is widely applied in orthopedics, such as in femoral head necrosis, joint replacement, and bone defects (Levine et al., 2006). However, its antibacterial properties remain controversial. In a large single-center study, Tokarski et al. found that porous Ta implants reduce infection rates during revision surgery and attributed this to the higher osseointegration ability, higher porosity, and inherent antibacterial properties of Ta (Tokarski et al., 2015). According to the competitive surface theory, osteoblasts are more likely to adhere and integrate on the surface, thereby reducing the dead space for bacterial colonization. In addition, porous Ta makes it difficult for organisms to contact and colonize. However, a subsequent analysis using the National Joint Registry in the English and Welsh databases did not reach a similar conclusion (Matharu et al., 2019). In *in vitro* studies and animal models, commercial porous Ta did not exhibit any inherent antibacterial properties (Chou et al., 2010; Harrison et al., 2017). Therefore, the lower infection rate of Ta is mostly related to its higher osseointegration ability.

At present, there are only a few reports on antibacterial Ta alloys. Spark plasma sintering was used to prepare a Ta-Cu alloy, and *in vitro* studies demonstrated its good corrosion resistance and antibacterial properties (Cui et al., 2017). In addition, Yang et al. investigated the antibacterial effect of Ta coatings and reported that the Ta nanomembrane mainly produces antibacterial effects by regulating innate immunity. It can enhance the phagocytosis of polymorphonuclear neutrophils, reduce the lysis of neutrophils, and enhance macrophages to promote the release of inflammatory cytokines, thereby forming a local host defense effect (Yang et al., 2019). Moreover, the micro-current generated between the Ta coating and the substrate consumes protons, resulting in increased ROS production in the environment around the implant and decreased bacterial ATP synthesis, which has an antibacterial effect (Zhang et al., 2019).

## Outlook

The biological functionalization of existing alloy systems is a promising method that can not only promote the integration of bone tissues, but also prevent infections. Compared with the simple modification of the coating, metal alloying can ensure that the material exhibits a strong and continuous antibacterial effect. However, there are still some challenges in the clinical applications of these novel antibacterial alloys. First, the addition of alloying elements must optimize the overall properties of the alloy, including the mechanical properties, corrosion behavior, wear resistance, antibacterial ability, and biological safety. Biological safety and mechanical properties are prerequisites for their material applications. Second, the underlying mechanism of antibacterial action needs to be further elucidated. Third, only a few studies have focused on the effect of antibacterial metal alloys on host immunity. Ag can impair the phagocytic response and induce atypical neutrophil death. This harmful effect on phagocytes may counteract the normal host immune response (Boraschi et al., 2017). Moreover, persistent low inflammation caused by the high activity of Ag may increase the incidence of IAI (Croes et al., 2018). In addition, Cu ions that can be used as a coating promoted the polarization of inflammatory macrophages both *in vivo* and *in vitro* and improved the killing effect of materials on MRSA (Huang et al., 2019; Liu et al., 2019). The relationship between copper antibacterial alloys in IAI and immunity has not yet been reported. Fourth, surface modification did not reduce the antibacterial properties of antibacterial alloys. Thus, a reasonable surface modification strategy can further improve the antibacterial ability of the alloy and other properties, such as the degradation speed. Finally, reasonable and accurate research design is a prerequisite for evaluating the properties and clinical application prospects of biomedical materials. The key points in the experimental design include, but are not limited to, the evaluation of antibacterial efficacy *in vivo*, the use of clinically isolated strains instead of standard strains, and the standardization of detection methods. Taking pure Mg and Mg alloys as examples, early studies have found favorable antibacterial effects *in vitro*, whereas some *in vivo* studies found that pure magnesium and magnesium alloys have significantly weaker antibacterial effects and even induce infections (Rahim et al., 2016; Brooks et al., 2018; Rahim et al., 2020). Therefore, the establishment of a precise *in vivo* model is undoubtedly critical for evaluating the real properties of materials. At present, there is still a lack of unified evaluation standards. Due to different detection methods or animal models, the heterogeneity of studies complicates the comparison between studies. In addition, clinically isolated and standard strains are not exactly the same. Most antibacterial studies of metals and alloys adopt standard strains instead of clinically isolated strains. The use of clinically isolated strains may better simulate real-world use, which is beneficial for clinical translation. More studies are needed to address these problems to improve the application of antibacterial alloys in the prevention and treatment of IAI.

## Conclusions

IAI treatment is still far from satisfactory owing to the complex interactions between bacteria, host cells, and implant materials. An in-depth understanding of the mechanism of IAI will help in the development of better materials. Incorporating antibacterial metals into existing materials is a promising strategy. This review discussed the mechanisms of action of antibacterial metals and alloys and provided a comprehensive summary of the current antibacterial metals and alloys for implant materials, paving the way for the development of novel options for the prevention and treatment of IAI.

## Author Contributions

BY and XQ conceived the general idea and provided critical revision and final approval of the manuscript. JJ and SZ conducted the literature study and wrote the draft manuscript. All authors contributed to the article and approved the submitted version.

## Funding

This work was supported by the National Natural Science Foundation of China (Grant No. 81972086 and 81672196); Shanghai “Rising Stars of Medical Talent” Youth Development Program (Youth Medical Talents – Specialist Program) (Grant No. 2019-72); National Key Research and Development Project of China (Grant No. 2020YFC1107500, and 2020YFC1107503); “Technology Innovation Action Plan” Key Project of Shanghai Science and Technology Commission (Grant No. 19411962800); Shanghai municipal education commission—Gaofeng clinical medicine grant support (Grant No. 20161423); Clinical Scientific innovation and Cultivation Fund of Renji Hospital Affiliated School of Medicine, Shanghai Jiaotong University (Grant No. PY2018-I-02); RENJI-NSFC Advancing Targeted Projects (RJTJ-JX-005).

## Conflict of Interest

The authors declare that the research was conducted in the absence of any commercial or financial relationships that could be construed as a potential conflict of interest.
